# The consequences of using advanced physical assessment skills in medical and surgical nursing: A hermeneutic pragmatic study

**DOI:** 10.3402/qhw.v11.32090

**Published:** 2016-09-06

**Authors:** Shelaine I. Zambas, Elizabeth A. Smythe, Jane Koziol-Mclain

**Affiliations:** Department of Nursing, Auckland University of Technology, Auckland, New Zealand

**Keywords:** Diagnostic reasoning, hermeneutics, interpretation, habit, physical assessment, pragmatism, advanced skill

## Abstract

**Aims and objectives:**

The aim of this study was to explore the consequences of the nurse's use of advanced assessment skills on medical and surgical wards.

**Background:**

Appropriate, accurate, and timely assessment by nurses is the cornerstone of maintaining patient safety in hospitals. The inclusion of “advanced” physical assessment skills such as auscultation, palpation, and percussion is thought to better prepare nurses for complex patient presentations within a wide range of clinical situations.

**Design:**

This qualitative study used a hermeneutic pragmatic approach.

**Method:**

Unstructured interviews were conducted with five experienced medical and surgical nurses to obtain 13 detailed narratives of assessment practice. Narratives were analyzed using Van Manen's six-step approach to identify the consequences of the nurse's use of advanced assessment skills.

**Results:**

The consequences of using advanced assessment skills include *looking for more*, *challenging interpretations*, and *perseverance*. The use of advanced assessment skills directs what the nurse looks for, what she sees, interpretation of the findings, and her response. It is the interpretation of what is seen, heard, or felt within the full context of the patient situation, which is the advanced skill.

**Conclusion:**

Advanced assessment skill is the means to an accurate interpretation of the clinical situation and contributes to appropriate diagnosis and medical management in complex patient situations.

**Relevance to clinical practice:**

The nurse's use of advanced assessment skills enables her to contribute to diagnostic reasoning within the acute medical and surgical setting.

## Summary Box

What this article contributes to the wider global clinical community?

- This research made visible the complex interplay of influencing factors in patient assessment and diagnosis.
- Interpretation of patient assessment findings is the advanced skill.
- More focus needs to be placed within nurse education on the interpretation of clinical assessment findings.

Acute care nurses are frequently described as the eyes and ears of the hospital, with their assessments considered critical to patient care and safety (Health and Disability Commissioner, 2009; Jacobs, Apatov, & Glei, 2007; Meyer & Lavin, 2005). In an effort to better prepare nurses for their role, the focus over the past few decades has been on teaching “advanced” assessment skills at both an undergraduate and postgraduate level. However, several authors have questioned the wide range of physical assessment skills taught (Birks, James, Chung, Cant, & Davis, 2014; Fennessey & Wittmann-Price, 2011; Giddens, 2007; Osborne, Douglas, Reid, Jones, & Gardner, 2015), particularly as many are rarely, if ever, used. While the number of skills most commonly used and barriers to assessment skill use have been explored, little has been done to explore the difference these skills make to patient outcomes.

A critique of the “systematic” physical assessment reveals that little is known about the value or impact of physical assessment skills on patient outcomes (Zambas, 2010). Osborne et al. (2015) argue that intervention studies are needed to link nursing assessment practice to patient outcomes. Linking nursing assessments to patient outcomes is challenging, however. Patient outcomes are varied and influenced by a myriad of factors and always involve a wider team than any one nurse. It is difficult to control for a single action or set of actions of a particular nurse. Furthermore, practice is seldom about any “one” action, for one thing leads to another, all within a complex interplay of influencing factors.

The pragmatic notion of consequence provided a way to explore the potential impact of advanced assessment skills on patient outcomes within these complexities. Doane and Varcoe (2005) argued, “every nursing moment is imbued with theory/practice and is thus an opportunity for theory development—for rethinking the ideas, assumptions, beliefs, and theories that govern our practice by examining the *consequences* of them” (p. 88, emphasis added). Nurses frequently review patient encounters with colleagues. In doing so, they describe their assessment findings, their immediate actions, and the consequences of those actions. It is possible to explore the relationship between advanced assessment skill use and patient outcomes through the nurse's experience of the consequences of using the skills in individual patient assessment encounters.

## Background

This study evolved out of an initial question posed by a charge nurse manager of an acute medical ward who asked, “What difference does learning these skills make to patient outcomes?” Much of the literature on the inclusion of physical assessment skills (inspection, auscultation, percussion, and palpation) into the nursing skill set is based on the belief that a more detailed physical assessment will improve patient outcomes (Douglas, Windsor, & Lewis, 2015; Yeung, Lapinsky, Granton, Doran, & Cafazzo, 2012). This assumption seems to apply to all nurses in all settings, not just to those for whom advanced assessment and diagnosis is a recognized part of their role description, such as nurse practitioners. While the inclusion of advanced physical assessment skills into general nursing practice is supported in principle, research suggests that use of the skills is complex. It is perplexing that the use of advanced assessment skills decreased with an increase in nursing experience (Osborne et al., 2015) and that level of education does not seem to influence skill use (Giddens, 2006). It is unclear how to interpret these findings. Explanations include increased workloads, more paperwork, and less time at the bedside as nurses gain more senior roles, as well as professional boundaries, clinical specialties, and ward environment affecting skill use (Birks et al., 2014; Edmunds, Ward, & Barnes, 2010; Fennessey & Wittmann-Price, 2011).

To date, little research has been conducted to identify the role of advanced physical assessment skills in improving patient outcomes. Patient outcomes are complex and multifaceted. They are affected by treatment and care provided, factors related to the patient, interpersonal aspects of care, and the setting or environment in which care is provided (Doran & Pringle, 2011). Because of the complexity of linking outcomes to specific nursing actions, the majority of research into nursing benefit has explored the relationship between nurse staffing levels (as an indicator of nursing care) and nurse sensitive outcomes such as pressure sores, falls, and adverse events (Doran & Pringle, 2011; Heslop & Lu, 2014). Although useful at an organizational level, Clarke (2006) points out that research at an organization level says little about the direct effects of specific nursing actions on patient outcomes.

Despite the lack of understanding of the role of advanced assessment skills in improving patient outcomes, much of the literature is critical of nurses not using the skills and contains arguments for why nurses should be integrating the skills into their practice. The language suggests an unquestioned belief in the benefits of advanced physical assessment. Words such as *should* and *why don't* are used in many of the articles written about physical assessment skills (Considine, 2005; Hogan, 2006; Schroyen, George, Hylton, & Scobie, 2005), and barriers to skill use continue to be explored (Douglas et al., 2014, 2015; Osborne et al., 2015). While a direct relationship between use of the skills and improved patient outcomes is frequently stated, no research is cited to support this relationship.

Giddens (2006) has suggested that many of the skills taught may not be needed for non-advanced clinical practice roles and raise the possibility of redundancy between the skills nurses are taught and those performed by junior doctors. This view is supported by more recent research. Edmunds et al. (2010) found that nurses selectively use assessment skills based on their clinical context and perceptions of role boundaries, permission, and cooperation. In exploring the barriers to advanced assessment skill use, Osborne et al. (2015) also found professional boundaries and the belief that specific assessment skills were designed for medical diagnosis to be significant.

To date, little research has been conducted to identify the role of advanced physical assessment skills in improving patient outcomes in general nursing practice. Although the theory of a comprehensive or more detailed physical assessment benefiting patients seems logical, the complexities of actual nursing practice leave its purpose ambiguous for a majority of nursing situations. With increasing demands on nursing resources and time, not to mention curriculum overload, the benefits and outcomes for patients of nurses learning and using these skills need to be demonstrated.

## Methods

### Aims

The aim of this study was to explore the consequences of the nurse's use of advanced assessment skills within the setting of acute medical and surgical wards. Medical and surgical wards were chosen as they are thought to most closely reflect the everyday enactment of nursing practice within acute care hospitals.

### Design

Both pragmatism and hermeneutics informed the design of this study and were chosen because of their ability to explore understanding and meaning within context (Charalambous, 2010; Doane & Varcoe, 2005). Pragmatism is a philosophy that has a focus on evaluating statements or ideas in terms of their usefulness or effectiveness in accomplishing a task. The connection between thinking and purpose is fundamental to pragmatism. John Dewey, one of the founders of pragmatism, believed that all human inquiry is tied exclusively to experience. He argued that we should consider all our knowledge as hypotheses to be tested in experience (Dewey, 1929/1958; Kloppenberg, 1996). His main concern was with what happens after an action is carried out (Polkinghorne, 2004) for it is the consequences of an action which give it meaning and justify its purpose. Pragmatism directs the inquirer to focus on the practical consequences of ideas, theories, and actions. The question “what are the consequences of the use of advanced assessment skills?” was a pragmatic one.

Pragmatism, however, does not specify a method. Hermeneutics was chosen to direct the method. Assessment is an act that nurses often do instinctively. They notice things without realizing they are looking; they simply do it. Hermeneutics is a methodology that seeks to language such ontological experience. It tries to uncover what is taken for granted. It was chosen as a way of helping nurses reveal consequences of both their conscious and unconscious use of advanced assessment skills. It sees understanding coming from the experience of a thing, in this case using the skills in actual practice. A pragmatic hermeneutic study allows for recognition of the experience of consequences of an individual's actions by interpreting stories of practice. This methodological approach allowed the complex interplay of influences to be revealed in the unfolding story. The goal of this research was to listen to stories as nurses related their experience of using the skills in specific patient encounters and then to interpret their stories of practice through the lens of consequence. This choice of methodology is described in more detail elsewhere (Zambas, Smythe, & Koziol-McLain, 2015).

### Ethical considerations

Ethics approval was obtained from Auckland University of Technology (ref no: 10/165) and the New Zealand Northern Regional Ethics Committee (NTY/10/04/033).

### Participants and recruitment

In order to identify the consequences of the use of advanced assessment skills, participants needed to be actively using the skills in their practice on medical and surgical wards. Purposeful sampling (Schneider, Whitehead, Elliot, LoBiondo-Wood, & Haber, 2007) was used to recruit registered nurses who met the following criteria:

- Used advanced assessment skills on a routine basis
- Were considered to demonstrate expert skill in patient assessment
- Had more than 1 year experience in their current practice setting (or similar)

Participants were recruited via charge nurse managers and clinical nurse educators from medical and surgical wards of both public and private hospitals within Auckland, New Zealand. Charge nurse managers and clinical nurse educators who observe nursing practice on a routine basis were felt to be in an ideal position to judge expert assessment practice. Expert assessment practice is about what nurses do and is reflected in practice that is considered safe, competent, and theoretically informed (Doane & Varcoe, 2008) by those in a position to judge this. Potential participants contacted the researcher to indicate interest in the study, to clarify the study aims and selection criteria, and to organize the first interview. Not all participants recognized themselves as having expert skill; however, they acknowledged their use of advanced assessment skills in their routine practice and the experience of being concerned about patients when other colleagues had missed cues.

Five participants were interviewed two to three times, resulting in 13 detailed stories of patient assessments. Participants were all female ranging in age from 36 to 54 and included nurses from medical and surgical wards, and adult and children's wards. Experience ranged from 3 years in practice to 30 plus years, and pay levels from competent to expert (competent×1, proficient×2, and expert×2). These levels reflected the nurse's current professional development, pay level, and overall level of practice–not their level of practice in relation to advanced assessment skills. Three nurses learned their advanced assessment skills through postgraduate education and two within the intensive care environment.

### Data collection

Allen (1995) argues that there is no “hermeneutic interview.” Instead, the interview process reflects the theory and questions being addressed. The researcher approaches the interview with an openness to be caught up in the play of the conversation in a manner that is in keeping with the study (Smythe, Ironside, Sims, Swenson, & Spence, 2008). It is always an interview about “something.” In this study, the interviews were about the nurse's assessment of a patient situation and their response to their assessment findings. Each interview began with the statement, “Tell me a story of when you think your use of advanced assessment skills made a difference for a patient you were caring for.” Prompts and further questions were based around the story about the patient situation and the nurse's assessment and response.

Prior to each interview, participants were asked to think of a story of practice in which they believed their use of advanced assessment skills made a difference to a patient they had cared for. Each story became an informal case presentation with the nurse describing assessment findings, thinking, and specific actions as they occurred over the course of the event. The interview style allowed frequent backtracking to clarify timelines and details within the case or ask the nurse to describe in more detail particular aspects of the assessment and interactions with the patient or other clinicians as a result of the assessment findings. There was an emphasis on identifying specific actions and communication resulting from the assessment. In all interviews, there was an attempt to identify what the nurse knew of the consequences of her actions for the patient.

Interviews occurred at a mutually agreed location, lasted between 60 and 90 min, and were digitally recorded. Two to three interviews were conducted with each participant to gain multiple stories from practice. Multiple interviews have the potential to stimulate a conscious process of reflection in action in relation to the phenomenon being investigated (Peden-McAlpine, 2000), heightening the participant's attention to the consequences of subsequent assessments.

### Data analysis

Interviews were transcribed verbatim. Individual stories were then rewritten into “narratives” using Caelli's (2001) process of constructing narratives. Narratives were returned to the participants for further clarification and to ensure accuracy and completeness. These became the texts used in the analysis. Pseudonyms were used in the narratives, and details of specific hospitals and wards omitted to maintain anonymity of participants, other members of the healthcare team, and the patients they were describing.

Analysis of each of the narratives was undertaken to identify themes using Van Manen's (1990) six-step approach: turning to the phenomenon, reflecting on thematic understanding, describing the phenomenon through writing and re-writing, remaining orientated to the question, and considering the parts and the whole. The emphasis within the analysis was on consequence, revealed through the story as it unfolded for the nurse participant. Annells (1999) criteria for establishing the quality of phenomenological research were used. The analysis was informed by Dewey (1922/2007) who argued that consequence includes the end result as well as the steps taken along the way (the means) to achieving the final end. The concept of means and ends was central to the analysis of the consequences of advanced assessment skill use, for the nurse cannot act until she has noticed. Trustworthiness of the research was judged using Annells (1999) criteria for establishing the quality of phenomenological research.

## Results

The stories of patient assessment encounters revealed assessment as it is played out throughout the course of an 8- or 12-h shift. The nurse has the opportunity, indeed obligation, to assess each time she is in visual contact with the patient, whether she is actively pursuing a task such as an initial assessment or simply notices something is not quite right while attending to some other patient care activity. For some of the nurses in this study, it was the initial assessment that caused them to be concerned; for others, it was one of the ongoing assessments that alerted the nurse to a problem. The findings are presented in a single vignette/case study. Smythe et al. (2016) argue for the use of a single story within hermeneutic research reporting in order to “let the story speak” to the reader. A single case allows for the subtle complexities in which signs and symptoms are revealed and the manner in which assessment skills contribute to recognition, interpretation, and response as the patient situation unfolds. In addition, the busyness and tensions within the context of that particular shift, ward, and clinical case are made visible.

### Consequence as looking for more

The consequences of using advanced assessment skills include looking closer and recognizing salient features. Looking closely not only helps the nurse see a problem, but also helps the nurse to gain an understanding of the nature of the problem. Maya tells a story of assessing a 4-year-old patient who was admitted to a pediatric medical ward with a diagnosis of viral illness, possibly gastroenteritis, 36 h previously. Because of a recent history of cancer, she is admitted under the oncology team rather than one of the medical teams. Maya begins her story:

She was handed over to me as “vomiting and a viral illness, possibly [gastroenteritis]”… When I was looking through her notes I just thought “oh well, she's just got a viral illness,” which is what the Oncology team had diagnosed. So I went and saw her and she was quite irritable. She was able to communicate with me, but she'd had a rough night and she was grumpy and had a sore head and was just feeling like crap. The first thing I did was take her pulse. She had the most unusual heart rate. It was really odd. It was basically fluctuating from 60 beats per minute up to 100 but real quick and very irregular. I was thinking “that is really strange.” That was a huge red flag for me. I carried on and did all my normal observations of her. She didn't want to eat or drink, she was on IV fluids. Everyone was thinking “that's okay, it's a viral illness. It's not a problem. She's just feeling miserable.” But I was concerned about the heart rate.

Although often considered the most basic and routine of nursing assessments, the recording of the pulse and blood pressure frequently provides the cue needed to recognize and begin exploring a problematic situation. In this story, Maya describes finding an abnormal pulse as a “huge red flag.” Her use of this phrase indicates her alertness, unease, and concern. The irregular low pulse is an unusual finding in a 4-year-old patient with a diagnosis of a viral illness. Maya recognized its unusualness and its “salience.” She acknowledges the role of habit in picking up an abnormal pulse:

I don't think you can see the signs if you're not assessing. When I walked in there at 8 o'clock there was a red flag… The other nurses weren't picking that up because they were taking her pulse with [an oxygen saturation] machine. They didn't notice that her heart rate was odd. Her pulse was fluctuating from 60, 70 up to 80, 90, 100, but a nurse just looking at the Sat machine is probably just going to look at the hundred. They're not going to ask “why does it drop down to 60.”

Maya believed she was able to pick up the unusual pulse because her habit is to palpate the pulse rather than rely on a monitor. While the oxygen saturation monitor provides a numerical reading of the pulse, fluctuations in recordings can be attributed to a number of other things. Thus, the irregular pulse may be overlooked. Maya's habit of always feeling for the pulse was the initial assessment act needed to recognize that something was not right.

Recognizing or “seeing” a concerning sign or symptom—a cue—stimulates further looking. Noting an unusual pulse set in motion actions to try to find a reason for it:

Once I recognised that the pulse wasn't right I was definitely reassessing this more often. And with a heart rate that was low and irregular, then the blood pressure was necessary …. No blood pressure had been taken on her previously because there wasn't any indication to do so.

Maya's recognition of the abnormal pulse triggered an assessment of blood pressure. The blood pressure recording is not a routine part of the vital sign measurements taken in young children. As Maya explained, there needs to be an indication for doing so. She described the significance of the blood pressure recording for this patient:

I'd been taking her blood pressure. I'd taken it earlier in the morning around ten and it was slightly elevated. I'd had a conversation with Mum at that point and she said, “Yeah this happened to her last time. Her blood pressure started to go up when she was really sick.” You get these little bits of information. I felt like something wasn't right but I wasn't 100 per cent sure, what it was.

There was no change in her neurological status. Even though I wasn't formally doing neuro obs… I was assessing all of it. I was always looking at her level of consciousness. I was checking her pupils and her muscle strength.

Maya revealed that the slightly elevated blood pressure, along with information from the mother, helped to reinforce her suspicion that something was not right. Each new cue helped to paint a picture of a concerning situation, but each on its own was not sufficient to help her identify exactly what it was that was causing her unease.

### Consequence as interpretation

The concerning situation is not necessarily obvious. Really seeing what is going on and recognizing a situation as concerning require interpretation of what is seen. Each individual assessment feature and each situation require interpretation, and thus, they are open to the potential for variation in how individuals interpret what they see and hear (Leder, 1990). Really seeing a problem requires interpretation of both the part and the whole. Maya's interpretation of the pulse was that it was unusual. Despite close monitoring and looking for other features, she was unable to make sense of it. She continued her narrative:

The difficult thing was because she was an outlier^1^ the oncology team weren't going to get down to her until about 11. She was under oncology because of her previous history, but she was on our ward because she had a viral illness. Finally, the doctors came down at about 10.30 or 11. The doctor wasn't exactly easy to work with. She was obviously in quite a bit of a rush. She was abrupt and wasn't really interested in hearing what I had to say. The first thing I said to her was that I was really concerned about the heart rate… The initial reaction from the doctor was, “Oh, yeah, that's okay. I'm not too worried about it.” And I was like, “Really?”

With hindsight and reflection, practitioners are often able to name the early signs of a problematic situation, but when they first feel, see, hear, or sense that something is not right they are often unsure if their concern is justified. By the time the doctor arrived on the ward, Maya's interpretation of the pulse was that it was not only unusual but inconsistent with the diagnosis this child had been given. She demonstrates her surprise when her communication of what she believed to be a worrying-finding was dismissed by the doctor. Maya tried to make sense of the doctor's lack of concern:

The doctor said, “Yeah, there could be a number of reasons why that is. I'm really busy. I've really got to go. I've got heaps of patients upstairs.” But I just didn't get where she was coming from in relation to a heart rate of 60. I kept asking “What would make her heart do that”? She didn't know. She was too busy. She had to go.

I said, “A, I'm not happy with the heart rate, and B, she's in a lot of pain.” So she said, “get an ECG.” I think that was just her way of getting out of the situation, but doing something as well. I was thinking “Great, excellent, fine, I'll do that. It will give me more information.”

Maya tried to engage the doctor in a discussion by questioning the suggestion that a number of things could be causing the abnormal heart rate. The initiation of a conversation served to keep her concern “in play” and demonstrated her need for a satisfactory conclusion. Genuine conversation is considered central to interpretation in clinical practice (Binding & Tapp, 2008). It is characterized by a stance of openness to the ideas offered by the other and by the awareness that the other may assist participants to revise their own partial understandings. In this situation, however, the doctor was busy and pushed for time.

The discrepancy between the doctor and Maya's interpretation is not an unusual occurrence. Brooks, LeBlanc, and Norman (2000) have suggested that contextual factors play a role in healthcare practitioners arriving at different interpretations of clinical situations. Maya had time to monitor the sign and mull it over. She was in and out of the room over the course of the morning, monitoring the pulse and then the blood pressure. She was also exploring the history of the illness with the child's parents. In contrast, the doctor had just arrived on the scene to “do rounds.” She would not have had time to form a reasoned opinion about the abnormal pulse when first notified of it. In addition, we do not know what background information the doctor had about the patient or her experience of viral illnesses in pediatric patients with a previous diagnosis of cancer.

Alongside the differing background understandings of the nurse and doctor, we know that this patient had an unusual history. Healthcare practitioners are taught to watch out for the “atypical” presentation. It is feasible that previous cancer in a young patient could predispose her to unusual physiological responses (Tolia & Smith, 2007). Individual signs need to be interpreted alongside other salient features of an illness.

In exploring the abnormal pulse as a sign of a problematic situation, Maya demonstrated how she moved from thinking “there is a problem” and reporting it, to trying to answer the question “what is the problem?” The consequences of her initial assessment and interpretation included looking for other salient features to help make sense of this usual sign. Maya's assessment actions were purposeful. In trying to figure out what was causing the illness, she was contributing to medical diagnostic reasoning. On a medical ward such as this, sharing one's interpretation of the clinical situation is often necessary in order to determine the most likely diagnosis and course of action.

The hermeneutic circle reminds us that interpretation occurs as a result of moving back and forth between the part and the whole. Maya might have been able to make her case of concern more strongly when she first reported it to the doctor if she had been able to pull together all of the information she had available to her, but she was not yet at that point. Although she recognized that the pulse was not normal, she was not sure what it was telling her. As Maya's story unfolded, the actions she had taken during the morning, which contributed to her sense of unease, but with which she was not yet able to articulate as a unified whole, are revealed.

Maya's recognition of the abnormal pulse led her to look for other signs. The slightly elevated blood pressure was sufficient to keep Maya alert to further cues. Critical and intelligent thought involves “the art of asking questions and of seeing what is questionable, of reflecting and contemplating, slowly weighing the strength and force of an argument, detecting what is salient …” (Fairfield, 2011, p. 95). Maya's thinking was influenced by her search for more clues, questioning, hearing what was said, and reflecting on the possibilities. She described her thinking:

It started with the headache. At first I thought “okay, a headache is feasible,” but then of course as I was building the relationship with the mum, you talk more and you get more information. I was listening to her sense that something was wrong, but also really listening to her story, to the history she was giving. What I got handed over was that she was continually vomiting, but she wasn't, she'd only had that Saturday night vomiting. And that she was having fevers, but she wasn't. She'd only had one fever. You can see how that information can change, where one fever becomes “fevers” and one evening of vomiting is interpreted as a gastro.

Maya described the information that gradually emerged to help her put together the pieces of this particular puzzle. She explored the history of the illness as she built rapport with the child's mother. The review of the history was not only a way of connecting with the family, demonstrating interest and “building rapport”; it was also information gathering in order to establish a cause of the illness. Maya really listened to this mother's story. It was a listening which heard the story differently from how it seems to have been heard by the doctors who made the initial diagnosis.

Has Maya's *listening* affected how she *heard* and interpreted this case? Baron (1990) argues that the patient story is “the mutual creation of the participants in the clinical encounter” (p. 28). He suggests that patients tell stories differently, depending on how questions are asked and what is asked, and this is a factor in arriving at different interpretations. Different questions will elicit different responses. In addition, patients may stress what they think is important. The simple telling of a story serves to emphasize some features in the mind of the teller and diminish others. Listeners too can alter a story by hearing what they want to hear.

Maya acknowledged how the interpretation of a viral illness might have been arrived at, particularly as the salient features of vomiting and fever were still in development when the diagnosis was made. But the vomiting and fever did not continue. Only the headache persisted. Thus, a story that looked like a viral illness yesterday no longer looks like a viral illness. Time itself has changed the story and its interpretation.

Maya also revealed a listening that included paying attention to the mother's sense that something was wrong. This listening is different from the history taking that is needed to establish a diagnosis. It is a listening that takes in the context of the illness and includes the mother's understanding of her child. This listening takes time and has a purpose beyond that of making a diagnosis and determining treatment. It hears more than has been said; it engages the listener. Binding and Tapp (2008) suggest that once we have truly heard, a connection is made between the listener and the listened to. Once this mother's concern was heard, it could not be ignored.

Maya did not arrive at her understanding of the case all at once or with absolute clarity. Her exploration of the background and interpretation evolve over time. She described exploring the history with the mother as a way of building rapport. But other opportunities also contributed to her interpretation of the whole. She explained:

… with the team coming in you're there listening to the mum's recollection to them and connections are being made. In the back of my mind constantly I was making connections and things weren't working out for me, and I was thinking “That's not right. That's not right. That's not right”…

Communicating, listening, and really thinking were critical to Maya's interpretation of the whole situation. Gadamer argues that “in order to be able to ask, one must want to know” (Gadamer, 1975/1989, p. 357). Asking relies on the knowledge that one does not know. Dewey echoes the importance of the need to know. He describes the attitude necessary for inquiry as that of actively *listening* rather than passively *hearing* (Talisse, 2000). Through active listening, a human bond is set up which culminates in the need to know. Maya's questioning reflects wanting to know. The consequence was an interpretation that was different from the medical team and ultimately more accurate.

### Consequence as perseverance

When the doctor was not able to offer an adequate explanation for the unusual pulse, Maya was not willing to let it rest. She pressed for action. She added the detail of a headache that was not responding to analgesia, finally getting some acknowledgement of her concern. She continued:

I didn't need her to order the ECG in order to do one. I was handing over some information that I wanted her to think about because it didn't seem right to me. But she didn't know either. It would have been great I guess if she had said, “Yeah, that's not normal. What else could there be that we're missing here?”

Maya communicated her concern by passing on information. She wanted the doctor, and by extrapolation the medical team, to rethink this child's diagnosis. Speaking and communicating contribute to thinking and to helping shape the interpretation of the situation (Habermas, 1984). Communicating concern is done in order to involve others in the mutual goal of problem solving.

While this narrative suggests poor assessment and judgment on the part of the doctor, this view is one that is privileged by hindsight. The doctor was looking at the information that Maya had presented but she was not able to make any more sense of it than Maya. Admittedly, she was distracted by other cases. Wright (2007) acknowledges that doctors “are potentially involved in several situations at once” (p. 156). They need to decide which of the concomitant situations should be attended to first. Decision making and clinical judgment is likely to be affected by numerous competing demands on time. Doctors are not alone in this juggle to prioritize time. All health practitioners need to prioritize the time they give to exploring the concerns of individual patients. We cannot know the specific issues this doctor was juggling on the morning that Maya presented her concern, or what else might have been influencing her interpretation of the headache and abnormal heart rate. All we know is that a patient with a current diagnosis of viral illness would have been prioritized alongside other acutely unwell patients. Maya acknowledged this:

I guess that is part of being a team; you continue to monitor and assess and then pull them in when you need them. Once they walk away I don't know what they're thinking; they might be just thinking about the next task whereas I was staying there. I was still looking after this child so I was still thinking about her. All day I was thinking about her; it's all I thought about …. She might have had other sicker children that she was worrying about herself that she had to go and see.

There is something about the nurse “being with” the patient and family throughout the day which demands attention. Proximity and the sense that “something wasn't right” meant this case was mulled over throughout the course of the 12-h shift. Eventually, enough pieces came together for Maya to recognize a serious problem. She described arriving at this point:

By about 2 I was done with waiting. I was done with it. Her blood pressure was going up, and her heart rate was staying on 60 and I knew that that was serious. This was a sign of elevated intracranial pressure. There was definitely something else happening. I said to Mum, “Look, I'm going to get the team down. We're going to work on this.”

I rang up the team. The doctor came down and I said to her, “Look at her blood pressure, look at her heart rate. She's got a really bad headache. You have to do something.” She started to freak a little bit, and that's when she ordered the urgent head CT. She finally looked at all these things together …

Wright (2007) describes the result of a successful inquiry as consensus among interested parties about the nature of the problem and the steps needed to yield a satisfactory resolution. This consensus was achieved when the signs of increased intracranial pressure became obvious, and Maya was finally able to convince the doctor that action was needed. While the story is unfolding, it can be difficult to identify the cause of the concern or unease, or to know where to look next. Maya's initial concern about one assessment finding triggered further looking, searching for clues as to the cause of the sign. She didn't stop looking. Her interpretation of the signs in relation to the whole context of the child's illness eventually came together as recognition of increased intracranial pressure. She described what happened once she made this connection:

I could see that I had to convince them that something else was going on. Once I had done that it was great because from there we were just going for it and we were communicating really well and we were working together …. We got the CT at about 4 o'clock …. It showed a massive brain hemorrhage.

Maya's assessment and interpretation of what she was seeing was the means through which a cerebral hemorrhage was diagnosed. Her continued search for specific signs and interpretation of each in relation to the whole situation eventually uncovered sufficient cues to gain the attention of the medical team and direct the next action. Maya was able to achieve this consequence because of her persistence and despite the initial lack of recognition by the medical team of what she felt was an incorrect diagnosis. Her assessment skill led to an interpretation that made a difference for this patient. It was the means to an accurate interpretation of the situation and appropriate medical management.

## Discussion

The process of nurses noticing, interpreting, and acting has been described previously as a model for how expert nurses think (Tanner, 2006). Recognizing patient change and deterioration through noticing is the surveillance role than some consider the essence of nursing (Dresser, 2012; Henneman, Gawlinski, & Giuliano, 2012; Meyer & Lavin, 2005). The nurse's role in arriving at an accurate interpretation and diagnosis of the clinical situation has received far less attention. Acting on problems requires health professionals to have an accurate understanding of the situation (Anderson, 2014). Individual patient assessments occur within the wider context of inter-professional practice and busy clinical environments. Alternative diagnostic hypotheses, the inability of all involved to see the same concerning features and inattention due to competing demands, provide additional challenges for health professionals in complex care environments. These external obstacles make conflict inevitable, particularly when practitioners arrive at different interpretations of the patient situation.

The frustrations nurses feel when their concern is not heard are not new (Benner, Tanner, & Chesla, 2009). Wright (2007), a medical practitioner, acknowledges the difficulty health practitioners have in mutually agreeing on assessment findings and identifying concerning patient situations. Agreement relies on both parties not only looking but seeing the full picture of the patient situation. The ability to agree on interpretations is complicated by the challenges of continually evolving signs and symptoms as well as differing levels of experience, training, and hierarchies. Most health practitioners would argue that they strive to collaborate over assessment and care decisions (Crosson, 2015), and yet nurses continue to report patient situations in which their valid interpretation is not listened to (Brier et al., 2015). As a result, nurses have begun to develop strategies for “packaging” the information that is relayed to doctors (Beckett & Kipnis, 2009; Brier et al., 2015; Curtis, Tzannes, & Rudge, 2011; Wacogne & Diwakar, 2010). In patient situations, where the nurse and other members of the healthcare team are in agreement, the use of communication aids such as SBAR (Situation, Background, Assessment, Recommendation or Response) seems appropriate. When the nurse's assessment and interpretation is not aligned with the medical practitioner, advanced assessment skill provides the stimulus and the rationale for looking further and for continuing to strive for shared understanding.

### Interpretation is the advanced skill

The interpretation of assessment findings is key to safe patient care. Health practitioners base their actions on their interpretation of a situation; however, signs and symptoms are at times ambiguous, even for expert practitioners (Brooks et al., 2000; Wears, 2009), and are therefore not always interpreted in the same way. People hear and interpret things differently. Some discrepancy is a result of the signs themselves; some is because signs can be transient in nature (lung sounds), while others (rash) are not easily discriminated even between experts. The assessment act itself is interpretive (Charalambous, 2010). Background understanding, experience, and beliefs shape what is heard or seen (LeBlanc, Brooks, & Norman, 2002; McCarthy, 2003). Each health professional ultimately arrives at their own interpretation, based on all that is within their vantage point. The patient's story, while being viewed within the same context and time, may be interpreted differently as each of the interpreters has slightly different vantage points.

Differences in the interpretation of signs and symptoms cause frustration and friction between health professionals (Benner et al., 2009; Manias & Street, 2001). Importantly, interpretations direct action and contribute to inaction. The initial interpretation of the situation will influence how seriously each sign or symptom is taken, how thoroughly it is investigated, and subsequent actions.

The focus on advanced assessment skill in this case study was in some ways the “elephant” in the room. As this narrative demonstrated, the skills, which enable nurses to know something is wrong and to pursue a specific course of action, are not necessarily “advanced.” The advanced part of their practice is the thinking that accompanies their assessment actions. It is the interpretation of the assessment findings in the search for a cause of the symptoms which makes Maya's assessment stand out as “advanced.” H. L. Dreyfus and S. E. Dreyfus (2005) make a distinction between crude skills such as the skill of placing the stethoscope on the chest and the subtle skills of interpreting the sounds heard. They argue, “subtle skill requires subtle discrimination” (p. 789). The real skill of advanced assessment is not the physical skills themselves; it is the skill of interpreting what is heard, seen, and felt within the full context of the individual patient situation. The use of specific skills provides essential information, but it is the interpretation which is the advanced skill.

### Nursing role in diagnostic reasoning and patient safety

The nurse in this case study kept this patient safe despite working in an environment that created numerous obstacles to safe patient care. These obstacles include patient loads that make it difficult to take the time needed to assess a patient more thoroughly or to follow up on concerns, cultural attitudes that value the doctor's knowledge, and voice above the nurse's and professional boundaries that identify medical diagnosis as outside the bounds of a registered nurse's practice. This is the environment of the medical and surgical ward. Cosby and Croskerry (2004) advocate for a “safety culture” within health care that “acknowledges safety as everyone's responsibility, promotes shared knowledge, and emphasizes teamwork” (p. 1344). The idea that diagnostic and treatment accuracy is a whole team responsibility is not widespread, yet is necessary to enable nurses to think differently about their role in preventing errors and maintaining patient safety.

A significant amount of literature exists around clinical reasoning and the nurse's role in interpreting deteriorating patient situations. The specific knowledge and skills needed to contribute to diagnostic and treatment decision is not as visible however, since this is “not the nurse's role” (Douglas et al., 2014). And while nurses are expected to act when they are concerned about the diagnosis or treatment a patient is receiving, little guidance is given for how to do this. The assumption in the literature around communication of concern and deterioration is that the nurse has not communicated well enough. There is little acknowledgement of the equal role of the doctor in listening to or hearing the message being conveyed. Matziou et al. (2014) found that nurses and doctors do not share the same views in relation to their communication and the nurses’ role in decision making. The most important barrier identified was that physicians do not recognize the nurses’ professional role.

Interpretation develops over time. The ability to “tell” all that has contributed to one's impression of a concerning patient situation is rarely acknowledged. Medical and nursing decision making is complex. Discerning the cause and appropriate actions in concerning patient situations requires a high level of clinical decision making. Signs and symptoms gradually reveal themselves; they are not always present waiting to be noticed or found like pieces of a ready-made puzzle (Baron, 1990; Peden-McAlpine & Clark, 2002). Presence enables snippets of information to be noticed and interpreted as they emerge. Seemingly, inconsequential bits of information percolate in the background, eventually taking shape alongside more concrete features of a patient's presentation until they form something that is recognizable and can be named. Accurate interpretation often requires subtle discrimination. The nurse who is present, constantly looking and interpreting, is in a prime position to discriminate between subtle features in order to grasp the situation and identify appropriate responses.

In addition, the healthcare environment itself is complex, with numerous demands placed on each member of the healthcare team. The ability of the nurse to use advanced skills to interpret the situation, to recognize the possibility of alternative diagnoses, to persevere, and to take responsibility for the outcome is critical to overcoming the systems and individual factors, which contribute to errors in clinical decision making. Assessment habit, which includes use of advanced skills, facilitates an accurate interpretation of the situation by the nurse and represents the culture of safety necessary to keep patients safe in complex healthcare settings (Page, 2004).

### Implications

Are advanced assessment, differential diagnosis, and diagnostic reasoning legitimate functions for the general staff nurse? Baid (2006) suggests, “a diagnosis can only be made if the professional is able to act upon the identified problem” (p. 1008). Nurses cannot legitimately diagnose medical illness or prescribe treatment, but they can act on the problems they identify via their recommendations. Identifying the problem and likely differential diagnoses enables them to initiate or recommend diagnostic tests or changes to treatment plans. They can make suggestions about potential diagnoses and treatments. They can escalate their concern through the nursing and medical hierarchy until their interpretation of the situation is heard. These are legitimate actions that nurses have control over; indeed, they are actions that are expected when the nurse recognizes a problematic patient situation (HDC, 2009).

### Limitations

All narratives tell one story in place of another (Kinsella, 2006). The consequences identified in this case reflect the nurse's perspective only. While the focus on consequences from the nurse's perspective fits with a hermeneutic study, the other actors’ voices have not been heard. Listening to their voices would provide a deeper understanding of the consequences of the nurse's assessment actions. The pragmatic lens of “consequence” was useful in revealing the value of advanced assessment skills. However, hermeneutics cautions our interpretation by asking, “What still lies hidden? What was closed down in coming up with this ‘interpretation?’ What else is to be thought?”

A further limitation relates to the nature of storytelling. Learning from past events is an imperfect process. Retrospective reviews “all suffer the limitation that they cannot faithfully reconstruct the context in which decisions were made and from which actions followed” (Croskerry, 2009). Fatigue, distractions, unconscious acts, and other patient responsibilities play a role in shaping what is noticed, and what is remembered when retelling an event. The context blurs with the passage of time. This limitation can be addressed by listening to and interpreting many stories of practice.

### Further research

Stories of practice from a wider range of nurses would support the understanding gained in this research. Both doctors’ and patients’ experiences of the consequences of nurses using advanced assessment skills also warrant investigation. Research suggests that doctors want and value a detailed description of patient problems (Weller, Barrow, & Gasquoine, 2011), but doctors experiences of the consequences of nurses using advanced assessment skills is unknown.

## Conclusion

Nurses make a significant contribution towards diagnostic and treatment decisions (Benner et al., 2009). Sometimes their contribution saves lives, as Maya's narrative revealed. This area of nursing practice has received less attention than other areas, such as the recognition of deterioration. This case study demonstrates the role of advanced assessment skills in a nurse's diagnostic reasoning research has shown that advanced assessment skills help nurses think differently. Maya in this study remained open to alternative diagnoses, other possibilities, to question, and to engage with and contribute to diagnostic and treatment decisions. The skills of advanced assessment shape what nurses look for and what they notice. Noticing causes them to interpret the situation in order to arrive at some understanding of its likely cause and the most appropriate response. Once understanding is achieved, the nurse is compelled to act. The tangible results of the use of advanced assessment skills on medical and surgical wards include going back to the doctor to ask for a reconsideration of clinical decisions, recommending diagnostic tests and treatment changes, and initiating specific treatments. The nurse's assessment, interpretation, and actions are means that contribute to achieving the best outcome for patients. This nurse saw what needed to be done, did what she had the authority to do, and worked to ensure those who did have authority did what was necessary. Her interpretation and consequent actions were the safety net that kept this patient safe.

## Footnotes

1 This patient is a child cancer survivor who has been placed on a medical ward.
